# The Role of Iron and Erythropoietin in the Association of Fibroblast Growth Factor 23 with Anemia in Chronic Kidney Disease in Humans

**DOI:** 10.3390/jcm9082640

**Published:** 2020-08-14

**Authors:** Bernhard Bielesz, Thomas Reiter, Fabian Peter Hammerle, Wolfgang Winnicki, Marija Bojic, Andreas Gleiss, Heidi Kieweg, Franz Ratzinger, Gere Sunder-Plassmann, Rodrig Marculescu

**Affiliations:** 1 Division of Nephrology and Dialysis, Department of Medicine III, Medical University of Vienna, 1090 Vienna, Austria; bernhard.bielesz@meduniwien.ac.at (B.B.); thomas.reiter@meduniwien.ac.at (T.R.); fabian@hammerle.me (F.P.H.); wolfgang.winnicki@meduniwien.ac.at (W.W.); marija.bojic@meduniwien.ac.at (M.B.); gere.sunder-plassmann@meduniwien.ac.at (G.S.-P.); 2 Center for Medical Statistics, Informatics, and Intelligent Systems, Medical University of Vienna, 1090 Vienna, Austria; andreas.gleiss@meduniwien.ac.at; 3 Department of Laboratory Medicine, Medical University of Vienna, 1090 Vienna, Austria; heidi.kieweg@chello.at (H.K.); franz.ratzinger@meduniwien.ac.at (F.R.)

**Keywords:** renal insufficiency, chronic, mineral metabolism, anemia

## Abstract

Anemia in chronic kidney disease (CKD) is an almost universal complication of this condition. Fibroblast growth factor 23 (FGF23), a key-player in mineral metabolism, is reportedly associated with anemia and hemoglobin levels in non-dialysis CKD patients. Here, we sought to further characterize this association while taking into account the biologically active, intact fraction of FGF23, iron metabolism, and erythropoietin (EPO). Hemoglobin, EPO, iron, and mineral metabolism parameters, including both intact and c-terminal-FGF23 (iFGF23 and cFGF23, respectively) were measured cross-sectionally in 225 non-dialysis CKD patients (stage 1–5, median eGFR: 30 mL/min./1.73m^2^) not on erythropoiesis stimulating agents or intravenous iron therapy. Statistical analysis was performed by multiple linear regression. After adjustment for eGFR and other important confounders, only cFGF23 but not iFGF23 was significantly associated with hemoglobin levels and this association was largely accounted for by iron metabolism parameters. cFGF23 but not iFGF23 was also associated with mean corpuscular hemoglobin (MCH) and mean corpuscular volume (MCV), again in dependence on iron metabolism parameters. Similarly, EPO concentrations were associated with cFGF23 but not iFGF23, but their contribution to the association of cFGF23 with hemoglobin levels was marginal. In pre-dialysis CKD patients, the observed association of FGF23 with hemoglobin seems to be restricted to cFGF23 and largely explained by the iron status.

## 1. Introduction

Anemia in chronic kidney disease (CKD) is a major complication of this condition. It is associated with increased morbidity and mortality and its prevalence increases as renal function declines, affecting the great majority of patients at late stages [1]. Among the pathophysiological mechanisms that are involved in anemia in CKD, iron, and erythropoietin deficiency play a prominent role [2].

Iron deficiency in non-dialysis CKD is thought to mainly result from elevated hepcidin levels and increased gastrointestinal blood loss [3,4]. The hepatic peptide hormone hepcidin is the master regulator of iron metabolism. It controls iron homeostasis by binding ferroportin, an iron exporting protein, thereby inhibiting iron flux from enterocytes, macrophages, and hepatocytes into the circulation [5]. The increased hepcidin levels in CKD are mainly attributed to the chronic inflammatory state and, to a lesser extent, to its decreased renal clearance [6].

The erythropoiesis-stimulating glycoprotein hormone erythropoietin (EPO) is mainly produced by peritubular renal fibroblasts. Renal tissue hypoxia leads to secretion of EPO via the induction of hypoxia-inducible factors (HIF) [7]. Anemia in CKD typically involves an inadequate EPO response to hemoglobin decline, resulting in inappropriately low EPO concentrations for the prevailing hemoglobin levels [8].

The osteocyte-secreted polypeptide fibroblast growth factor 23 (FGF23) is a key regulator of phosphate homeostasis [9]. FGF23 increases as renal function deteriorates and it is associated with adverse outcomes [10]. FGF23 circulates in blood both in its intact, biologically active form, and as inactive fragments resulting from specific enzymatic cleavage [11]. The current FGF23 literature is based on immunoassays that are specific for the former (iFGF23) and on immunoassays targeting exclusively the C-terminus, which react with both intact FGF23 and its c-terminal cleavage products (cFGF23). The results of these two types of assays have been shown to diverge in several circumstances [12,13].

Recently, FGF23 has been linked to physiological processes beyond phosphate homeostasis, including iron metabolism, EPO secretion, and erythropoiesis. Iron deficiency and acute administration of recombinant EPO increased cFGF23, but not iFGF23 serum concentrations in humans [13,14]. In mice, the daily administration of recombinant EPO induced FGF23 gene expression in bone marrow erythroid cells and increased serum concentrations of cFGF23 after 24 h and of iFGF23 after four days [14]. Conversely, FGF23 has been shown to inhibit erythropoiesis in mice, partially by suppressing EPO secretion [15,16].

In CKD patients, associations of FGF23 serum concentrations with hemoglobin levels and the presence of anemia have been described [17,18,19]. However, in these studies, only cFGF23 was measured and no analysis of potential underlying mechanisms was undertaken. Here, we attempted to further characterize the observed association of FGF23 with hemoglobin in CKD by taking into account both cFGF23 and the biologically active iFGF23, as well as the biomarkers of iron metabolism, including hepcidin, and EPO levels.

## 2. Experimental Section

### 2.1. Patients

We included patients that were enrolled in a CKD cohort established at the Division of Nephrology and Dialysis, Department of Medicine III, Medical University of Vienna, comprising patients of all six eGFR categories (Supplemental Figure S1)

The exclusion criteria for this cohort were age less than 18 years, pregnancy, and history of kidney transplantation. Serum and plasma samples were collected in a biobank repository. Clinical and demographic data were retrieved from the participating patients and from medical charts at the time of inclusion. The study protocol was approved by the ethics committee of the Medical University of Vienna (project number EK2012/2016), adhered to the Declaration of Helsinki, and all of the patients provided written informed consent.

### 2.2. Laboratory Methods

We used laboratory tests that are certified for in vitro diagnostics, as follows: Red blood count, hemoglobin, erythrocyte indices (Sysmex XE-5000, Sysmex, Kobe, Japan), total calcium, inorganic phosphate (iP), albumin, protein, creatinine, intact PTH, iron, transferrin, ferritin, IL-6 (Cobas 8000, Roche Diagnostics, Mannheim, Germany), 25-hydroxy-vitamin D, (25(OH)D), 1,25-dihydroxy-vitamin D (1,25(OH)2D), intact FGF23 (iFGF23) (Liaison XL, DiaSorin, Saluggia, Italy), erythropoietin (EPO) (Immulite 2000 XPI, Siemens Healthineers, Erlangen, Germany), and soluble transferrin-receptor (sTFR) (BN II System, Siemens Healthineers, Erlangen, Germany). For c-terminal FGF23 (cFGF23), the Immutopics Human FGF-23 (C-Term) ELISA (Quidel, San Diego, CA, USA), for Klotho the Soluble alpha-Klotho ELISA (IBL, Imuno-Biological Laboratories Co., Ltd., Fujioka, Japan), for Hepcidin the Human Hepcidin Quantikine ELISA (R&D Systems Inc., Minneapolis, MN, USA) were used according to the manufacturers’ instructions. The estimated glomerular filtration rate (eGFR) was determined according to the CKD-EPI formula. All of the analyses were performed in ISO 15189 accredited clinical laboratories of the Department of Laboratory Medicine at the Medical University of Vienna.

### 2.3. Statistics

The categorical variables are described by counts and percentages. Continuous variables are described by means and standard deviations if approximately normally distributed and by medians and interquartile ranges otherwise. Linear regression models were used to quantify the effect of continuous markers on continuous outcome parameters. Variables exhibiting right-skewed distributions were log-transformed before used in a model. For a log-transformed independent variable, the estimated regression coefficient thus quantifies the effect of doubling this variable. For a log-transformed dependent variable, the regression coefficient was back-transformed to quantify a multiplicative effect on the original variable’s scale. The R^2^ values are reported to quantify the proportion of variability in the dependent variable that is explained by an independent variable or a set of variables, i.e., for comparing their prognostic importance. Multiple regression models were used to adjust investigated influences for the potential confounders albumin, gender, eGFR, and presence of diabetes mellitus type II. 95% confidence intervals are provided for all reported estimates. Residual distributions were graphically checked. None of the potentially influential observations exhibited a relevant impact on the results upon deletion in the adjusted models.

The amount of blood that was drawn at study inclusion was not completely uniform because of variable tube filling at the venipuncture procedure leading to insufficient sample material for some analyses. Therefore, we assume that missing data are missing at random and are not associated with the unmeasured values. Accordingly, each model is based on complete cases with respect to the variables involved. The total numbers of values used for calculation are reported for each variable in Supplemental Table S1.

*p*-values that were below 0.05 were regarded as statistically significant. No correction for multiple testing was done due to the exploratory character of this study. All of the calculations were done using SAS version 9.4 (SAS Enterprise, SAS Institute Inc., Cary, NC, USA).

## 3. Results

### 3.1. Study Cohort

We included 225 patients with CKD stage G1 to G5 in this study (median eGFR 30 mL/min./1.73m^2^, eGFR range 2.5–152 mL/min./1.73m^2^). Native kidney disease was diabetic nephropathy: 26; vascular nephropathy: 41; polycystic kidney disease: 13; glomerulonephritis: 67; interstitial nephritis: 6; other (HIV associated nephropathy, tumor nephrectomy, systemic vasculitis, congenital ureteral disease and reflux, drug toxicity, cardio-renal-secondary to heart failure, Alport syndrome): 35; unknown: 37. Table 1 indicates demographic details and the results of biomarker measurements.

### 3.2. Predictors of Hemoglobin Concentrations

c-terminal and intact FGF23 both show a statistically significant influence on hemoglobin in the unadjusted analysis (Table 2). However, only the association with c-terminal FGF23 is significant after adjustment for renal function, gender, albumin, and the presence of diabetes mellitus II (base model), although the effect is attenuated (adjusted *β* = −0.28; *p* = 0.001 vs. unadjusted *β* = −0.65; *p* < 0.001). Multiple parameters of mineral metabolism, iron metabolism, EPO, and inflammatory markers show a statistically significant effect on hemoglobin in the unadjusted analysis. However, in the adjusted base model, only the influences of calcium, phosphate, serum iron, transferrin saturation, hepcidin, and EPO remain significant beside cFGF23 (Table 2).

### 3.3. Association of Iron Metabolism Biomarkers, EPO, and Inflammation with Hemoglobin Concentrations

We added selected sets of parameters to account for mediation via iron, EPO, and inflammation pathways to assess differential pathways implicated in erythropoiesis that might be involved in a proposed cFGF23 hemoglobin axis (Table 3). Adding indices of iron metabolism (iron model: ferritin, transferrin, transferrin saturation, hepcidin, and serum iron) largely displaces the influence of cFGF23 on hemoglobin (*β* = –0.15; *p* = 0.126 vs. *β* = –0.28; *p* = 0.001). Adding EPO to the base model (EPO model) only has a modest impact on the effect of cFGF23 on hemoglobin, which remains statistically significant (*β* = −0.22; *p* = 0.012 vs. *β* = −0.28; *p* < 0.001). Finally, adding the parameters of inflammation (C-reactive protein, interleukin 6) does not influence this association at all. No significant association of iFGF23 with hemoglobin is found in any of these additional models.

The associations of phosphate and calcium with hemoglobin are largely independent of iron status, EPO, and inflammation biomarkers (Supplemental Table S2).

### 3.4. Association of cFGF23, Phosphate, and Total Calcium with Iron Status, EPO, and Inflammation biomarkers

For further investigation of mediation, we tested the influence of cFGF23, iFGF23, calcium, and phosphate on the potentially mediating parameters discussed above (Table 4). Consistent with the previous findings, cFGF23 displays statistically significant influence on the iron metabolism parameters transferrin saturation, serum iron, ferritin, and hepcidin, as well as EPO. Additionally, an association with interleukin 6 is observed. Again, no significant associations are found for iFGF23.

No other statistically significant relationships of phosphate or calcium with any of the potential mediators under assessment are apparent, except for an association of phosphate with EPO (Supplemental Table S3).

### 3.5. Association of cFGF23 with Erythrocyte Indices

We next looked at the erythrocyte indices mean corpuscular hemoglobin (MCH) and mean corpuscular volume (MCV), given the strong involvement of iron parameters in the association of cFGF23 with hemoglobin. Consistently, we found that cFGF23, but not iFGF23, phosphate, or calcium significantly influences MCH and this association is abolished in the iron, but not in the EPO or inflammation adjusted model (Supplemental Table S4). Similar findings were evident for MCV after additional adjustment for folic acid and cobalamin (vitamin B12) levels (Supplemental Table S5).

## 4. Discussion

In this cross-sectional study, we aimed to further characterize the role of FGF23 in anemia in CKD. For this purpose, we analyzed both total cFGF23 and bioactive iFGF23, biomarkers of iron metabolism, including hepcidin, inflammation, and EPO, in a cohort of non-dialysis CKD patients with predominantly moderate to severe renal function impairment (median eGFR 30 mL/min./1.73m^2^). Our main findings are as follows:

(1) We confirm the previously described association of total cFGF23 with hemoglobin in non-dialysis CKD patients, however (2) such an association does not hold true for the bioactive iFGF23 in an adjusted model. (3) Biomarkers of iron metabolism and, to a lesser extent, EPO appear to largely explain the association of cFGF23 with hemoglobin.

The association of FGF23 with anemia or hemoglobin has been previously described in three independent studies of non-dialysis CKD patients [17,18,19]. However, all of them analyzed total cFGF23 only and did not account for markers of iron metabolism or EPO. cFGF23 assays measure bioactive iFGF23 and c-terminal FGF23 fragments. The latter result from proteolytic cleavage of iFGF23 by furin proteases directly in its producing cells, mainly osteocytes [20]. Although the overall physiologic significance of this process is currently not understood, it seems to be specifically regulated and contributes to the control of systemic FGF23 action, given that the resulting (c-terminal and n-terminal) fragments appear to lack inherent FGF23 activity [11]. However, c-terminal fragments of FGF23 have been shown to antagonize iFGF23 function by acting as a competitive blocking peptide in mice [16,21].

We show that the reported associations of cFGF23 with hemoglobin do not extend to the intact form of FGF23 in the adjusted analysis. Similar divergences have been previously described in several settings. In iron deficient renal transplant recipients cFGF23, but not iFGF23, appeared to mediate the association of iron deficiency with mortality [22]. Red cell distribution width was associated with cFGF23, but not iFGF23 in CKD and congestive heart failure patients [23]. EPO was shown to induce total cFGF23 out of proportion to iFGF23 [24].

One of the specific triggers of FGF23 cleavage by proteolysis appears to be iron deficiency, which contributes to the pathophysiology of anemia in CKD. Wolf et al. [13] reported elevated cFGF23, but not iFGF23 levels in kidney-healthy iron deficient women that predominantly decreased after iron administration. It was proposed that iron deficiency increases the secretion of iFGF23 that is rapidly cleaved under normal circumstances. Meanwhile, these findings have been corroborated at the mechanistic level in an experimental animal study [25].

We see a robust correlation of iron indices with cFGF23 (Table 4). Total cFGF23, but not biologically active iFGF23, is associated with hemoglobin levels as well as MCH and MCV. This association is blunted after accounting for iron metabolism parameters suggesting that the observed inverse association of cFGF23 with hemoglobin levels in non-dialysis CKD patients relates to a large part to iron metabolism. Lower iron stores act both by directly promoting anemia and increasing cFGF23 by stimulating iFGF23 production and cleavage. Interestingly, there is also evidence for a reverse causal relationship, i.e., that FGF23 contributes to iron deficiency by increasing hepcidin levels as indicated by experimental iFGF23 blockage [16].

We observed a significant influence of cFGF23, but not iFGF23 on EPO serum levels (Table 4). Furthermore, EPO attenuated the statistical interaction of cFGF23 and hemoglobin, albeit not to the same degree as iron (Table 3). The exact mechanisms as to how FGF23 might be involved in the hematopoietic system, are unclear. However, increased erythropoiesis, as indicated by a higher number of erythrocytes and hematopoietic stem cells in peripheral blood and bone marrow has been reported in FGF23 knockout mice [15]. Moreover, the activation of HIF target genes, higher serum EPO levels, and increased EPO mRNA in kidney and liver were found in this animal model [15]. The same group has shown in the murine 5/6 nephrectomy model that FGF23 contributes to anemia in CKD by suppressing EPO, inducing iron deficiency, and increasing apoptosis [16]. On the other hand, the exogenous administration of EPO has been demonstrated to stimulate FGF23 mRNA expression in bone and increase cFGF23 and iFGF23 serum levels in humans and rodents [14,26,27].

The associations of phosphate and calcium with hemoglobin remain robust in adjusted analyses in agreement with previous reports [28,29] (Supplemental Table S2). However, none of our tested models affected these associations to a relevant degree (Supplemental Table S3).

We note a significant direct association of cFGF23 with IL-6, which is consistent with previous data that inflammatory markers associate with FGF23 levels in CKD [30,31]. We do not observe significant associations of CRP or IL-6 with hemoglobin in the adjusted analysis. Likewise, these parameters do not affect the association of hemoglobin with cFGF23 in our cohort. However, the overall degree of inflammation was, at most, modest, as 129 subjects out of 225 (57%) had a CRP of less than 1 mg/dl and IL-6 levels of less than 7 pg/mL.

Jointly, our data render a quite consistent picture of FGF23 in the clinical context of anemia in CKD, in which its interaction with iron metabolism holds a prominent rank. The invariant divergence between cFGF23 and iFGF23 in terms of associations with all studied indices of hematopoiesis and iron metabolism is striking. Interestingly, it was shown that higher doses of iron supplementation in hemodialysis patients improve cardiovascular outcomes [32]. It is tempting to speculate that this could be mediated by decreased FGF23 transcription and cleavage as a consequence of improving iron status. More studies are needed in order to clarify the impact of iron supplementation on intact FGF23 and its fragments in CKD patients. There is an increasing number of immunoassays, both for the c-terminal and intact FGF23 protein, approved for routine clinical diagnostic use in the European Union. Awareness of the impact of iron and EPO metabolism on test results has important clinical implications.

The main limitation of our study is its cross-sectional design, which precludes definitive causal or mechanistic conclusions. This needs to be addressed in future studies.

However, our study has several strengths: The study was conducted in humans, in a representative patient cohort of a large outpatient clinic comprising the full range of renal function impairment not on dialysis. We excluded patients on ESA or intravenous iron therapy, as these interventions could have confounded the FGF23 measurements. Moreover, we wanted to avoid potential cross-reactivity with the EPO assay for the various ESA preparations. We measured FGF23 using both intact and c-terminal assays and performed a detailed analysis of a wide range of parameters that are involved in iron metabolism and inflammation as well as endogenous EPO, which, to the best of our knowledge, has never been analyzed before in the context of FGF23′s role in anemia in human CKD.

## 5. Conclusions

We demonstrate that cFGF23 levels, which comprise both c-terminal fragments and the intact form, rather than the hormonally active iFGF23, are associated with hemoglobin levels in CKD patients. Iron metabolism and, to a lesser extent, EPO seem to account for this association, in agreement to previous experimental data.

## Figures and Tables

**Table 1:** Demography and biomarkers of 225 study participants according to the stage of CKD.

	CKD I	CKD II	CKD IIIa	CKD IIIb	CKD IV	CKD V
n	21	26	26	42	80	30
Sex (M/F)	14/7	11/15	15/11	33/9	48/32	23/7
Age (years)	35 (13.4)	49 (16.4)	61 (11.3)	63 (12.7)	62 (13.5)	57 (15.2)
BMI (kg/m^2^)	27 (4.9)	27 (5.9)	28 (6.5)	28 (4.8)	27 (5.6)	27 (5.9)
Erythrocytes (T/L)	4.9 (0.6)	4.7 (0.4)	4.4 (0.6)	4.4 (0.5)	4.1 (0.5)	3.7 (0.6)
Hemoglobin (g/dL)	15 (1.8)	14 (1.6)	13 (1.6)	13 (1.6)	12 (1.5)	11 (1.6)
Hematocrit (%)	42.6 (4.9)	40.8 (4.3)	38.7 (4.5)	38.8 (4.2)	35.8 (4.3)	32.2 (5.1)
MCV (fl)	87 (4.1)	88 (5.5)	89 (4.4)	89 (5.9)	88 (6.3)	87 (4.8)
MCH (pg)	30 (1.6)	29 (2.1)	30 (1.6)	29 (1.9)	29 (2.2)	29 (1.6)
MCHC (g/dL)	34 (0.9)	34 (1.2)	33 (0.9)	33 (1.1)	33 (1.1)	33 (1.3)
RDW (%)	13 (0.8)	14 (2.4)	14 (1.4)	14 (1.2)	14 (1.7)	15 (1.5)
Total calcium (mmol/L)	2.4 (0.2)	2.5 (0.1)	2.4 (0.2)	2.4 (0.1)	2.3 (0.2)	2.3 (0.2)
Alb-corr calcium (mmol/L)	2.3 (0.1)	2.4 (0.1)	2.4 (0.1)	2.3 (0.1)	2.3 (0.2)	2.3 (0.3)
Phosphate (mmol/L)	1.0 (0.9–1.1)	1.0 (1.0–1.1)	1.1 (0.9–1.2)	1.1 (1.0–1.2)	1.2 (1.1–1.3)	1.6 (1.–1.8)
Creatinine (mg/dl)	0.86 (0.79–0.98)	0.99 (0.86–1.17)	1.34 (1.19–1.5)	1.88 (1.69–2.04)	2.66 (2.27–3.07)	5.05 (4.2–6.04)
Protein (g/L)	70.1 (9.2)	70.6 (7.1)	70.7 (7.1)	70.6 (6.5)	68.2 (6.3)	69.3 (7.3)
Albumin (g/L)	44 (6.6)	43 (4.6)	41 (3.9)	42 (4.1)	41 (4.1)	41 (4.2)
PTH (pg/mL)	22 (20–28)	35 (29–57)	45 (36–73)	64 (48–86)	107 (80–164)	191 (10–323)
eGFR (ml/min/1.73m²)	101 (95–107)	74 (69–78)	51 (47–56)	35 (32–38)	23 (18–26)	11 (1–13)
cFGF23 (RU/mL)	71 (66–127)	107 (78–161)	126 (105–187)	221 (158–382)	299 (195–450)	820 (54–1837)
iFGF23 (pg/mL)	58 (55–68)	68 (51–79)	95 (81–110)	119 (100–153)	172 (132–264)	924 (40–4676)
Klotho (pg/mL)	627 (385–1408)	660 (614–699)	644 (497–826)	649 (552–803)	522 (415–622)	480 (413–549)
CRP (mg/dL)	0.2 (0.1–0.3)	0.1 (0.1–0.6)	0.4 (0.2–0.6)	0.4 (0.2–0.9)	0.3 (0.1–0.8)	0.8 (0.4–1.8)
IL-6 (pg/mL)	1.5 (1.5–2.4)	2.2 (1.5–5.8)	4.3 (3–6.1)	6.0 (4.5–10.8)	6.4 (3.5–12.9)	9.8 (6.6–16.5)
25(OH)D (nmol/L)	57 (43–70)	51 (26–81)	58 (35–79)	59 (43–80)	43 (26–72)	36 (25–53)
1,25(OH)2D (pmol/L)	60 (41–74)	52 (38–63)	36 (30–46)	34 (24–42)	26 (19–34)	22 (16–32)
EPO (mU/mL)	6.3 (4.4–9.9)	8.5 (6.9–14.7)	9.7 (7.4–16.7)	13.0 (8.8–20.5)	9.5 (7–14.7)	10.8 (7–15.2)
Hepcidin (pg/mL)	17353 (7789–28894)	24374 (9501–43820)	29067 (18155–41327)	27956 (19053–38164)	31306 (16513–56586)	48667 (32297–91577)
Ferritin (µg/L)	98 (46–147)	111 (37–191)	116 (70–216)	103 (55–190)	104 (50–174)	119 (73–242)
Transferrin (mg/dL)	263.8 (40.1)	273.7 (53.4)	249.5 (48.5)	252.2 (50.4)	240.2 (39.5)	217.3 (30.2)
TSAT (%)	28.9 (11.7)	22.5 (8.8)	25.1 (9.3)	20.5 (7.5)	22.2 (9.0)	21.5 (6.9)
Serum iron (µg/dL)	112.0 (67–132)	84.5 (72–105)	86.0 (58–109)	69.0 (52–79)	69.0 (51–90)	61.0 (53–72)
sTFR (mg/L)	1.2 (1–1.5)	1.3 (1.1–1.9)	1.3 (1.2–1.6)	1.5 (1.3–1.8)	1.4 (1.1–1.7)	1.4 (1.1–1.8)
PK (mg/g)	137.0 (52–547)	214.0 (84–1225)	256.0 (82–1201)	318.0 (129–1233)	748.5 (229–1758)	1717.0 (1073–2354)

BMI: body mass index; MCV: mean corpuscular volume; MCH: mean corpuscular hemoglobin; MCHC: mean corpuscular hemoglobin concentration; RDW: red cell distribution width; alb-corr calcium: albumin-corrected calcium; BUN: blood urea nitrogen; PTH: parathyroid hormone; eGFR: estimated glomerular filtration rate (CKD-EPI formula); cFGF23: c-terminal Fibroblast Growth Factor 23; iFGF23: intact Fibroblast Growth Factor 23; CRP: C-reactive protein; IL-6: Interleukin 6; 25(OH): 25-hydroxy-Vitamin D3; 1,25(OH)2D: 1, 25-dihydroxy-Vitamin D3; EPO: erythropoietin; SD: standard deviations; TSAT: transferrin saturation; sTFR: soluble transferrin receptor; PK: protein creatinine ratio; shown are mean and standard deviation (SD) for normally distributed variables or median and quartiles (Q1–Q3) for variables with skewed distribution. Reference ranges: total calcium: 2.2–2.65 mmol/l; phosphate: 0.91–1.45 mmol/l; magnesium: 0.66–1.07 mmol/l; creatinine men: 0.70–1.20 mg/dl, women: 0.50–0.90 mg/dl; protein: 64–83 g/L; albumin: 35–52 g/L; cFGF23: 0.8 pmol/L (median); iFGF23: 23.2–95.4 pg/mL; PTH: 15–65 pg/mL; 25(OH)D: 75-250 nmol/L; 1,25(OH)2D: 19.9–79.3 pg/mL; RDW: 11–16%; TSAT: 16–45 %; CRP: < 0.5 mg/dl; IL-6: < 7 pg/mL; serum iron (>18 yrs): 33–193 g/dl; sTFR (>18 years): 0.76-1.76 (mg/L); PK: < 150 mg/g.

**Table 2:** Dependence of hemoglobin on biomarkers of mineral metabolism, iron metabolism, EPO concentrations, and inflammation biomarkers.

	Dependent Variable					
	Hemoglobin (g/dL)					
	Unadjusted			Adjusted Base Model		
Biomarker	Beta	*p*	R^2^	Beta	*p*	R^2^
cFGF23 (RU/mL) *	−0.65 (−0.78/−0.51)	**<0.001**	0.29	−0.28 (−0.44/−0.11)	**0.001**	0.49
iFGF23 (pg/mL) **	−3.26 (−4.01/−2.51)	**<0.001**	0.25	−1.04 (−2.1/0.03)	0.057	0.45
Klotho (pg/mL) *	1.04 (0.39/1.69)	**0.002**	0.07	0.30 (−0.26/0.87)	0.289	0.43
Total calcium (mmol/L)	4.35 (3.09/5.6)	**<0.001**	0.17	2.15 (0.91/3.39)	**<0.001**	0.48
Alb-corr calcium (mmol/L)	3.21 (1.67/4.75)	**<0.001**	0.07			
Phosphate (mmol/L) *	−2.48 (−3.11/−1.84)	**<0.001**	0.21	−0.75 (−1.42/−0.07)	**0.032**	0.46
PTH (pg/mL) *	−0.65 (−0.83/−0.46)	**<0.001**	0.18	−0.14 (−0.36/0.07)	0.191	0.46
25(OH) (nmol/L) *	0.46 (0.16/0.75)	**0.003**	0.04	0.06 (−0.19/0.30)	0.658	0.45
1,25(OH)2D (pmol/L) *	0.89 (0.59/1.19)	**<0.001**	0.14	0.02 (−0.29/0.32)	0.911	0.45
Serum iron (µg/dl) *	1.41 (0.96/1.85)	**<0.001**	0.18	0.74 (0.33/1.16)	**<0.001**	0.46
Ferritin (µg/L) *	0.09 (−0.1/0.28)	0.37	0	0.07 (−0.09/0.22)	0.404	0.45
Transferrin (mg/dl)	0.01 (0/0.02)	**0.004**	0.05	0 (−0.01/0.01)	0.905	0.42
TSAT (%) *	0.96 (0.51/1.41)	**<0.001**	0.09	0.56 (0.18/0.94)	**0.004**	0.45
sTFR (mg/L) *	0.31 (−0.3/0.92)	0.321	0.01	0.47 (0/0.95)	0.052	0.43
Hepcidin (pg/mL) *	0.03 (-0.1/0.15)	0.695	0	0.14 (0.04/0.24)	**0.007**	0.47
EPO (mU/mL) *	−0.53 (−0.79/−0.28)	**<0.001**	0.07	−0.34 (−0.53/−0.15)	**<0.001**	0.48
CRP (mg/dl) *	−0.16 (−0.3/−0.02)	**0.026**	0.02	0.05 (−0.07/0.17)	0.407	0.45
IL 6 (pg/mL) *	−0.47 (−0.64/−0.29)	**<0.001**	0.12	0 (−0.16/0.17)	0.961	0.45

Regression coefficients (beta) and confidence intervals with hemoglobin as dependent variable for respective markers from unadjusted linear regression models (left columns) and from regression models adjusting for albumin, gender, eGFR, and presence of Diabetes mellitus type II (adjusted base model, right columns). R^2^ values for the adjusted base model refer to adjustment variables plus the respective marker (adjustment variables alone achieve R^2^ = 0.45). * indicates that calculations were performed with binary-log-transformed marker values such that beta quantifies the effect of doubling the marker (otherwise beta quantifies the effect of one additional unit). ** indicates that calculations were performed with double log-transform, i.e., binary log of the binary log of iFGF23, such that beta quantifies the effect of squaring the value of iFGF23. PTH: parathyroid hormone; cFGF23: c-terminal Fibroblast Growth Factor 23; iFGF23: intact Fibroblast Growth Factor 23; 25(OH)D: 25 hydroxy-vitamin D_3_; 1,25(OH)_2_D: 1, 25 dihydroxy-vitamin D_3_; CRP: C-reactive protein; IL-6: Interleukin 6; TSAT: transferrin saturation; sTFR: soluble transferrin receptor; EPO: erythropoietin; eGFR: estimated glomerular filtration rate (CKD-EPI formula).

**Table 3:** Influence of cFGF23 and iFGF23 on hemoglobin after adjusting for biomarkers of iron metabolism, EPO concentrations and inflammation biomarkers.

	Dependent Variable					
	Hemoglobin (g/dL)					
	* cFGF23 (RU/mL)			** iFGF23 (pg/mL)		
Model	Beta	*p*	R^2^	Beta	*p*	R^2^
Base model	−0.28 (−0.44/−0.11)	**0.001**	0.49	−1.04 (−2.1/0.03)	0.057	0.45
Iron model	−0.15 (−0.34/0.04)	0.126	0.51	−0.89 (−1.99/0.22)	0.115	0.51
EPO model	−0.22 (−0.38/−0.05)	**0.012**	0.51	−0.83 (−1.89/0.22)	0.121	0.48
Inflammation model	−0.28 (−0.45/−0.11)	**0.001**	0.49	−1.01 (−2.09/0.08)	0.068	0.45

Regression coefficients (beta) and confidence intervals with hemoglobin as dependent variable for respective markers from regression models adjusting for albumin, gender, eGFR, and presence of Diabetes mellitus type II (Base model; only adjustment variables: R^2^ = 0.45); Base model plus serum iron, ferritin, transferrin, transferrin saturation and hepcidin (Iron model; only adjustment variables: R^2^ = 0.50); Base model plus EPO (EPO model; only adjustment variables: R^2^=0.48); Base model plus CRP and IL-6 (Inflammation model; only adjustment variables: R^2^ = 0.45). R^2^ values refer to adjustment variables plus the respective marker. * indicates that calculations were performed with binary-log-transformed marker values such that beta quantifies the effect of doubling the marker (otherwise beta quantifies the effect of one additional unit). ** indicates that calculations were performed with double log-transform, i.e., binary log of the binary log of iFGF23, such that beta quantifies the effect of squaring the value of iFGF23. cFGF23: c-terminal Fibroblast Growth Factor 23; iFGF23: intact Fibroblast Growth Factor 23; EPO: erythropoietin; CRP: C-reactive protein; IL-6: Interleukin 6; eGFR: estimated glomerular filtration rate (CKD-EPI formula).

**Table 4:** Influence of cFGF23 and iFGF23 on biomarkers of iron metabolism, EPO concentrations and inflammation biomarkers.

	Independent Variable					
	* cFGF23 (RU/mL)			** iFGF23 (pg/mL)		
Dependent Variable	exp(Beta)	*p*	R^2^	exp(Beta)	*p*	R^2^
TSAT (%)	0.89 (0.85/0.94)	**<0.001**	0.17	0.95 (0.68/1.31)	0.744	0.06
Serum iron (µg/dl)	0.92 (0.88/0.96)	**<0.001**	0.25	1.04 (0.78/1.39)	0.802	0.18
Ferritin (µg/L)	0.89 (0.81/0.99)	**0.032**	0.20	1.09 (0.56/2.14)	0.795	0.17
Hepcidin (pg/mL)	0.73 (0.63/0.85)	**<0.001**	0.22	0.72 (0.26/1.97)	0.519	0.15
EPO (mU/mL)	1.17 (1.08/1.27)	**<0.001**	0.11	1.57 (0.93/2.66)	0.09	0.05
CRP (mg/dl)	1.01 (0.88/1.16)	0.863	0.17	0.63 (0.27/1.49)	0.291	0.18
IL 6 (pg/mL)	1.11 (1.01/1.22)	**0.029**	0.32	1.19 (0.64/2.23)	0.575	0.31

Back-transformed regression coefficients (exp(Beta)) and confidence intervals for the dependent variables are indicated in the line headings. The respective independent variable (marker) is indicated by column heading (cFGF23, iFGF23, phosphate, total calcium). Regression models were adjusted for albumin, gender, eGFR, and presence of Diabetes mellitus type II (Base model). Independent variables: * indicates that calculations were performed with binary-log-transformed independent variables such that Beta quantifies the effect of doubling the independent variable (otherwise Beta quantifies the effect of one additional unit). ** indicates that calculations were performed with double log-transform, i.e., binary log of the binary log of iFGF23, such that beta quantifies the effect of squaring the value of iFGF23. Dependent variables: all of the dependent variables have been binary-log transformed and subsequently estimates back-transformed, such that the given coefficient (exp(Beta)) quantifies a multiplicative effect of the independent variable (column heading) on the dependent variable (line heading); e.g., effect of cFGF23 on TSAT: doubling cFGF23 translates into an average reduction of TSAT by 11% (0.89xTSAT); effect of calcium on TSAT: one unit increase in calcium translates into a non-significant average reduction of TSAT by 20% (0.80xTSAT). cFGF23: c-terminal Fibroblast Growth Factor 23; iFGF23: intact Fibroblast Growth Factor 23; TSAT: transferrin saturation; EPO: erythropoietin; CRP: C-reactive protein; IL-6: Interleukin 6; eGFR: estimated glomerular filtration rate (CKD-EPI formula).

## Supplementary Materials

The following are available online at https://www.mdpi.com/2077-0383/9/8/2640/s1, Figure S1: Patient disposition.; Table S1: Total sample number per variable (excluding missing values); Table S2: Influence of phosphate and total calcium on hemoglobin after adjusting for biomarkers of iron metabolism, EPO concentrations, and inflammation biomarkers; Table S3: Influence of phosphate and total calcium on biomarkers of iron metabolism, EPO concentrations, and inflammation biomarkers; Table S4: Influence of cFGF23, iFGF23, phosphate, and total calcium on mean corpuscular hemoglobin (MCH) after adjusting for biomarkers of iron metabolism, EPO concentrations, and inflammation biomarkers; Table S5: Influence of cFGF23, iFGF23, phosphate, and total calcium on mean corpuscular volume (MCV) after adjusting for biomarkers of iron metabolism, EPO concentrations, and inflammation biomarkers.
