# Supplementary material for: The Role of Iron and Erythropoietin in the Association of Fibroblast Growth Factor 23 with Anemia in Chronic Kidney Disease in Humans
Source: J Clin Med. 2020 Aug 14;9(8):2640. doi: 10.3390/jcm9082640 (PMC7463779; doi:10.3390/jcm9082640)
Supplement: Supplementary file 1 [file jcm-09-02640-s001.pdf]

Journal of
Clinical Medicine

MDPI

## Supplementary Material

**Supplemental Figure 1.** Patient disposition.

AKI, acute kidney injury; CKD, chronic kidney disease; ESA, erythropoiesis stimulating agent

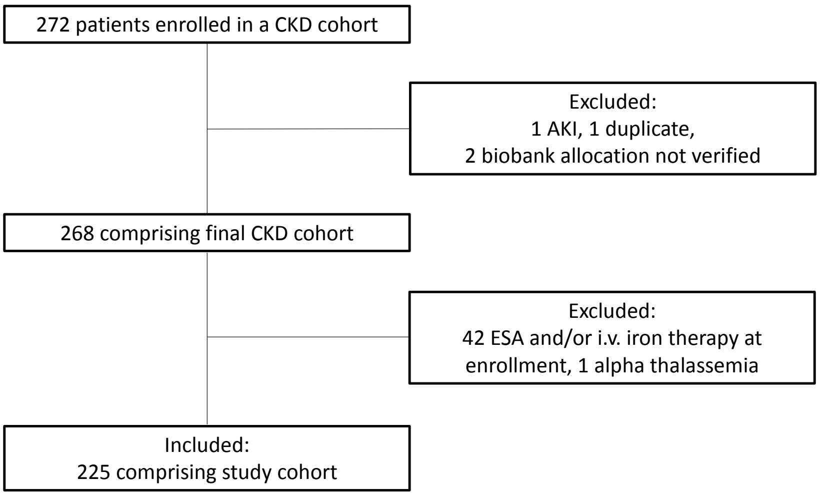


**Supplemental Table 1:** Total sample number per variable (excluding missing values)

| Variable | total number |
|---|---|
| 25(OH)D (nmol/l) | 219 |
| 1,25(OH)2D (pmol/l) | 218 |
| PTH (pg/mL) | 221 |
| IL-6 (pg/mL) | 222 |
| EPO (mU/mL) | 222 |
| Klotho (pg/mL) | 127 |
| cFGF23 (RU/mL) | 221 |
| iFGF23 (pg/mL) | 221 |
| Hepcidin (pg/mL) | 222 |
| eGFR (ml/min/1.73m$^2$) | 225 |
| Total calcium (mmol/l) | 225 |
| Alb-corr calcium (mmol/l) | 219 |
| Phosphate (mmol/l) | 225 |
| CRP (mg/dl) | 222 |
| Erythrocytes (T/l) | 223 |
| Hemoglobin (g/dl) | 223 |
| Hematocrit (%) | 223 |
| MCV (fl) | 223 |
| MCH (pg) | 223 |
| MCHC (g/dl) | 223 |
| sTFR (mg/L) | 178 |
| TSAT (%) | 177 |
| Ferritin (µg/L) | 222 |

| | |
|---|---|
| Transferrin (mg/dl) | 177 |
| Serum iron (µg/dl) | 177 |
| RDW (%) | 223 |
| Creatinine (mg/dl) | 225 |
| PK (mg/g) | 217 |
| Protein (g/l) | 208 |
| Albumin (g/l) | 219 |

MCV: mean corpuscular volume; MCH: mean corpuscular hemoglobin; MCHC: mean corpuscular hemoglobin concentration; RDW: red cell distribution width; alb-corr calcium: albumin-corrected calcium; BUN: blood urea nitrogen; PTH: parathyroid hormone; eGFR: estimated glomerular filtration rate; cFGF23: c-terminal Fibroblast Growth Factor 23; iFGF23: intact Fibroblast Growth Factor 23; CRP: C-reactive protein; IL-6: Interleukin 6; 25(OH): 25-hydroxy-Vitamin D3; 1,25(OH)2D: 1, 25-dihydroxy-Vitamin D3; EPO: erythropoietin; TSAT: transferrin saturation; sTFR: soluble transferrin receptor; PK: protein creatinine ratio.

**Supplemental Table 2:** Influence of phosphate and total calcium on hemoglobin after adjusting for biomarkers of iron metabolism, EPO concentrations, and inflammation biomarkers.

Dependent variable
Hemoglobin (g/dl)

| | *Phosphate (mmol/l) | | | Total calcium (mmol/l) | | |
|---|---|---|---|---|---|---|
| Model | Beta | p | $R^2$ | Beta | p | $R^2$ |
| Base model | -0.75 (-1.42/-0.07) | **0.032** | 0.46 | 2.15 (0.91/3.39) | **<0.001** | 0.48 |
| Iron model | -0.76 (-1.51/-0.02) | **0.045** | 0.51 | 1.95 (0.65/3.24) | **0.003** | 0.53 |
| EPO model | -1.08 (-1.75/-0.4) | **0.002** | 0.51 | 2.34 (1.14/3.54) | **<0.001** | 0.52 |
| Inflammation model | -0.76 (-1.45/-0.06) | **0.033** | 0.46 | 2.11 (0.86/3.37) | **0.001** | 0.48 |

Regression coefficients (beta) and confidence intervals with hemoglobin as dependent variable for respective markers from regression models adjusting for albumin, gender, eGFR, and presence of Diabetes mellitus type II (Base model; only adjustment variables: $R^2$=0.45); Base model plus serum iron, ferritin, transferrin, transferrin saturation and hepcidin (Iron model; only adjustment variables: $R^2$=0.50); Base model plus EPO (EPO model; only adjustment variables: $R^2$=0.48); Base model plus CRP and IL-6 (Inflammation model; only adjustment variables: $R^2$=0.45). $R^2$ values refer to adjustment variables plus the respective marker. * indicates that calculations were performed with binary-log-transformed marker values such that beta quantifies the effect of doubling the marker (otherwise beta quantifies the effect of one additional unit). EPO: erythropoietin; CRP: C-reactive protein; IL-6: Interleukin 6; eGFR: estimated glomerular filtration rate (CKD-EPI formula).

**Supplemental Table 3:** Influence of phosphate and total calcium on biomarkers of iron metabolism, EPO concentrations, and inflammation biomarkers.

Independent variable

| | *Phosphate (mmol/l) | | | Total calcium (mmol/l) | | |
|---|---|---|---|---|---|---|
| Dependent variable | exp(Beta) | p | $R^2$ | exp(Beta) | p | $R^2$ |
| TSAT (%) | 1.04 (0.84/1.29) | 0.723 | 0.06 | 0.80 (0.54/1.17) | 0.246 | 0.07 |
| Serum iron (µg/dl) | 0.97 (0.80/1.18) | 0.788 | 0.17 | 0.91 (0.64/1.28) | 0.572 | 0.18 |
| Ferritin (µg/L) | 1.15 (0.75/1.76) | 0.514 | 0.17 | 1.24 (0.56/2.71) | 0.594 | 0.17 |

| | | | | | | |
|---|---|---|---|---|---|---|
| Hepcidin (pg/mL) | 1.82 (0.97/3.44) | 0.063 | 0.16 | 1.48 (0.46/4.81) | 0.512 | 0.15 |
| EPO (mU/mL) | 0.58 (0.42/0.8) | **0.001** | 0.09 | 1.48 (0.8/2.73) | 0.215 | 0.05 |
| CRP (mg/dl) | 0.77 (0.45/1.32) | 0.339 | 0.18 | 1.6 (0.59/4.38) | 0.358 | 0.18 |
| IL 6 (pg/mL) | 0.69 (0.46/1.02) | 0.060 | 0.33 | 0.86 (0.41/1.77) | 0.671 | 0.32 |

Back-transformed regression coefficients (exp(Beta)) and confidence intervals for the dependent variables are indicated in the line headings. The respective independent variable (marker) is indicated by column heading (phosphate, total calcium). Regression models were adjusted for albumin, gender, eGFR, and presence of Diabetes mellitus type II (Base model). Independent variables: * indicates that calculations were performed with binary-log-transformed independent variables such that Beta quantifies the effect of doubling the independent variable (otherwise Beta quantifies the effect of one additional unit). Dependent variables: all of the dependent variables have been binary-log transformed and subsequently estimates back-transformed, such that the given coefficient (exp(Beta)) quantifies a multiplicative effect of the independent variable (column heading) on the dependent variable (line heading); e.g., effect of calcium on TSAT: one unit increase in calcium translates into a non-significant average reduction of TSAT by 20% (0.80 × TSAT). TSAT: transferrin saturation; EPO: erythropoietin; CRP: C-reactive protein; IL-6: Interleukin 6; eGFR: estimated glomerular filtration rate (CKD-EPI formula).

**Supplemental Table 4:** Influence of cFGF23, iFGF23, phosphate, and total calcium on mean corpuscular hemoglobin (MCH) after adjusting for biomarkers of iron metabolism, EPO concentrations, and inflammation biomarkers.

| | *cFGF23 (RU/mL) | | | **iFGF23 (pg/mL) | | |
|---|---|---|---|---|---|---|
| Model | Beta | p | $R^2$ | Beta | p | $R^2$ |
| Base model | -0.29 (-0.51/-0.06) | **0.013** | 0.13 | -0.42 (-1.89/1.06) | 0.576 | 0.10 |
| EPO model | -0.31 (-0.54/-0.07) | **0.011** | 0.14 | -0.43 (-1.92/1.06) | 0.571 | 0.11 |
| Iron model | -0.07 (-0.32/0.18) | 0.587 | 0.29 | -0.23 (-1.67/1.21) | 0.752 | 0.29 |
| Inflammation model | -0.27 (-0.51/-0.04) | **0.021** | 0.14 | -0.4 (-1.88/1.09) | 0.599 | 0.12 |

| | *Phosphate (mmol/l) | | | Total calcium (mmol/l) | | |
|---|---|---|---|---|---|---|
| Model | Beta | p | $R^2$ | Beta | p | $R^2$ |
| Base model | -0.04 (-0.97/0.88) | 0.927 | 0.10 | 0.69 (-1.03/2.41) | 0.431 | 0.11 |
| EPO model | -0.11 (-1.07/0.85) | 0.815 | 0.11 | 0.71 (-1.01/2.44) | 0.416 | 0.11 |
| Iron model | 0.02 (-0.96/0.99) | 0.975 | 0.29 | 0.67 (-1.04/2.39) | 0.438 | 0.29 |
| Inflammation model | -0.12 (-1.06/0.83) | 0.808 | 0.12 | 0.69 (-1.03/2.42) | 0.430 | 0.12 |

Regression coefficients (Beta) and confidence intervals with mean corpuscular hemoglobin (MCH) as dependent variable for indicated independent variables (column heading) adjusting for albumin, gender, eGFR, and the presence of Diabetes mellitus type II (Base model); Base model plus ferritin, transferrin, transferrin saturation, hepcidin, serum iron (Iron model); Base model plus EPO (EPO model); Base model plus CRP, IL-6 (Inflammation model). $R^2$ values refer to adjustment variables plus the respective marker. * indicates that calculations were performed with binary-log-transformed marker values such that Beta quantifies the effect of doubling the marker (otherwise Beta quantifies the effect of one additional unit). ** indicates that calculations were performed with double log-transform, i.e., binary log of the binary log of iFGF23, such that beta quantifies the effect of squaring the value of iFGF23.cFGF23: c-terminal Fibroblast Growth Factor 23; iFGF23: intact Fibroblast Growth Factor 23; EPO: erythropoietin; CRP: C-reactive protein; IL-6: Interleukin 6.

**Supplemental Table 5:** Influence of cFGF23, iFGF23, phosphate, and total calcium on mean corpuscular volume (MCV) after adjusting for biomarkers of iron metabolism, EPO concentrations, and inflammation biomarkers.

| | *cFGF23 (RU/mL) | | | **iFGF23 (pg/mL) | | |
|---|---|---|---|---|---|---|
| Model | Beta | p | $R^2$ | Beta | p | $R^2$ |
| Base model | -0.95 (-1.79/-0.11) | **0.027** | 0.11 | -3.18 (-8.24/1.87) | 0.215 | 0.08 |
| EPO model | -0.99 (-1.85/-0.13) | **0.024** | 0.11 | -3.2 (-8.29/1.89) | 0.216 | 0.08 |
| Iron model | -0.58 (-1.42/0.27) | 0.181 | 0.23 | -3.62 (-8.42/1.18) | 0.138 | 0.23 |
| Inflammation model | -1.04 (-1.89/-0.19) | **0.017** | 0.14 | -3.68 (-8.8/1.43) | 0.157 | 0.10 |

| | *Phosphate (mmol/l) | | | Total calcium (mmol/l) | | |
|---|---|---|---|---|---|---|
| Model | Beta | p | $R^2$ | Beta | p | $R^2$ |
| Base model | -2.12 (-5.62/1.37) | 0.232 | 0.08 | 2.24 (-4.39/8.86) | 0.505 | 0.07 |
| EPO model | -2.22 (-5.84/1.39) | 0.226 | 0.08 | 2.25 (-4.47/8.97) | 0.508 | 0.07 |
| Iron model | -1.88 (-5.32/1.57) | 0.282 | 0.22 | 3.45 (-3.2/10.1) | 0.306 | 0.22 |
| Inflammation model | -2.07 (-5.64/1.5) | 0.253 | 0.10 | 2.32 (-4.35/9) | 0.492 | 0.09 |

Regression coefficients (Beta) and confidence intervals with mean corpuscular volume (MCV) as dependent variable for indicated independent variables (column heading) adjusting for albumin, gender, eGFR, the presence of Diabetes mellitus type II, and in addition for folic acid and vitamin B12 (Base model); Base model plus ferritin, transferrin, transferrin saturation, hepcidin, serum iron (Iron model); Base model plus EPO (EPO model); Base model plus CRP, IL-6 (Inflammation model). $R^2$ values refer to adjustment variables plus the respective marker. * indicates that calculations were performed with binary-log-transformed marker values such that Beta quantifies the effect of doubling the marker (otherwise Beta quantifies the effect of one additional unit). ** indicates that calculations were performed with double log-transform, i.e., binary log of the binary log of iFGF23, such that beta quantifies the effect of squaring the value of iFGF23. cFGF23: c-terminal Fibroblast Growth Factor 23; iFGF23: intact Fibroblast Growth Factor 23; EPO: erythropoietin; CRP: C-reactive protein; IL-6: Interleukin 6.
